# Device closure in adults with atrial septal defect in Shiraz, a single center registry

**DOI:** 10.15171/jcvtr.2016.07

**Published:** 2016-03-15

**Authors:** Mohammad Ali Ostovan, Javad Kojuri, Pooyan Dehghani, Vida Razazi, Alireza Moarref

**Affiliations:** ^1^Cardiovascular Research Center, Cardiology Department, Shiraz University of Medical Sciences, Shiraz, Iran; ^2^Master of Management, School of Management and Information, Shiraz University of Medical Sciences, Shiraz, Iran

**Keywords:** Atrial Septal Defect, Transcatheter Closure, Surgical Closure, ASD Devices, Complications

## Abstract

***Introduction:*** Successful closure of atrial septal defect (ASD) improves patients’ functional class and exercise capacity. In this study we evaluate the safety and feasibility of percutaneous device closure of ASDs.

***Methods:*** Two hundred fifty six patients with significant ASD according to our criteria were enrolled. The patients were treated using nitinol wire mesh transcatheter devices. Complications were followed for a median of 2.5 years.

***Results:*** Success rate was 98.4% with 3 unsuccessful cases and a mean hospital stay of 1.007 ± 0.0004 days. Complication rate was 7.42%. Size of the right ventricle (RV) annulus was significantly decreased 24 hours after intervention (P = 0.005).

***Conclusion:*** The present report demonstrates that transcatheter closure of ASD is safe and effective.

## Introduction

Atrial septal defect (ASD) has a prevalence of 6%-10% of congenital heart diseases.^1^ Ostium secundum ASDs are often suitable for percutaneous repair however with stricter selection criteria than surgical repair. Anomalous pulmonary venous connection, additional intracardiac anomalies, proximity of the defect to atrioventricular valves, coronary sinus or systemic venous drainage, deficient rims over a large portion of the circumference of the defect and very large ASDs (>35-40 mm) usually precludes the use of this technique.^2^ Percutaneous device closure can be done safely in experienced hands with rare complications including device embolization, atrial arrhythmias, stroke, chamber perforation and thrombus formation in less than 1% of the patients.^3^ Successful defect closure improves functional class and exercise capacity of the patient.^4^

Surgical closure was the treatment of choice for hemodynamically significant ASDs in the past. In 1976, first successful percutaneous closure in human was performed by King et al.^5^ This method became an interesting substitute for surgical closure as it obviates the need for thoracotomy, open heart surgery with cardioplegia and cardiopulmonary bypass. Other advantages include shorter hospital stays, less postoperative pain, avoidance of surgical scars and reduction in the incidence of dysrhythmias due to absence of atrial scar.^6^ There are several reports of successful ASD closures using different devices with few complications in short and long term follow- up.^7-9^

Although there are some reports of transcatheter ASD device closure In Iran,^10-12^ none had such a large number of patients as ours. The aim of this study is evaluation of the safety and feasibility of percutaneous device closure of ASDs.

## Materials and Methods

This study was a prospective study on 256 consecutive patients who were referred to Shiraz heart centers from 2003-2014. Patients had final echocardiographic diagnosis of ASD with Qp/Qs ratio of more than 1.5 and/or enlarged right ventricle (RV). The size and shape of the defect, number of defects, and association to mitral and aortic valve were evaluated. Important rims, malalignment and aneurysm of the septum were documented.

Our inclusion criteria were ASD secundum with maximum diameter of 34 mm; rims except aortic rims of at least 5 mm and dimensions of total septal length not smaller than left atrial (LA) disk of the chosen device.

Patients aged more than 40 years underwent diagnostic coronary angiography prior to the procedure. The patients were treated using nitinol wire mesh transcatheter devices for closure of the defect. The procedure was performed under local anesthesia and the guidance of transesophageal echocardiography (TEE) in 248 patients and under the guidance of transthoracic echocardiography in the remaining eight. Heparinization with 100 U/kg of unfractionated heparin was done.

ASD was crossed with a multipurpose or cournand catheter and a 0.035″ guidewire. Then the catheter was positioned in the right upper pulmonary vein and the guidewire was exchanged with the 0.035″ super stiff wire in order to give the support required for large delivery sheaths. In those cases which were done under guidance of TEE, balloon sizing of ASD was done with stop flow technique.

Four types of devices were used in this study including Amplatzer (AGA Medical Corporation, USA), Starway (Starway Medical Technology, Inc., China), Lifetech (Lifetech Scientific Co., Ltd., China) and Occlutech (Occlutech GmbH, Germany).

After successful closure, the patients were put on dual antiplatelet therapy of aspirin (160 mg) and Plavix (75 mg) for 3 months. It was followed by aspirin (80 mg) therapy for at least 6 months.

Complications were followed and recorded. Transcatheter closure is associated with all the general risks in any interventional cardiac catheterization procedures such as contrast reactions, vessel or cardiac perforation. The most common complication of femoral vein access is hematoma which may be increased with larger venous sheaths needed for the larger devices.

Short term complications included post procedure large groin hematoma, air emboli with transient ST elevation, transient pericardial effusion, transient recurrent paroxysmal supraventricular tachycardia, transient frequent, premature ventricular beats requiring therapy, transient atrial fibrillation, residual shunt (Qp/Qs) less than 1.5 and transient ischemic attack. Long term complications included death, clot over LA disc, and late device malposition.

Follow-up electrocardiography (ECG) and transthoracic echocardiography were performed in 24 hours and 2 weeks after the procedure. TEE was done after 6 months.

Detection of arrhythmia was based on continuous monitoring during and after the procedure for 24 hours and taking 12-lead ECG. Residual shunt was defined as the significant color flow through the device or passage of more than five bubbles of contrast (agitated saline) during TEE. The patient were followed for a median duration of 2.5 years (1-10 years).

Data were analyzed by the SPSS software version 15.0. We used statistical descriptive techniques and paired *t* test. P value <0.05 was considered statistically significant.

## Results

Two hundred fifty six cases (164 female, 92 male) were enrolled with the mean age of 26.7 ± 7.6 years. Concomitant coronary artery disease (CAD) was present in 2 patients (0.7%).

Two patients were post-surgical cases with incomplete repair. Four types of devices were used with Occlutech as the most common (Table 1). Mean defect size was 21 ± 4 mm and mean device size was 24 (range: 15-34 mm). Procedure was done with transthoracic guidance in 8 patients (3.1%).

**Table 1:** Device Types Used in the Study

**Device Type**	**Number (%)**
Amplatzer ( AGA)	48 (18.75)
Starway	50 (19.53)
Lifetech	5 (1.95)
Occlutech	153 (59.76)

The number of difficult anomalies was 74 (28.9%) and the most common ones were deficient aortic rim (43 [16.79%]), aneurysmal septum (10 [3.9%]) and large ASD (balloon occlusion diameter >30) (7 [2.73%]) (Table 2).

**Table 2:** Difficult Anatomies

**Difficult Anatomies**	**Number**
Deficient aortic rim	43
Deficient posterior rim	5
Deficient aortic and posterior rim	3
Deficient superior rim	1
Aneurysmal septum	10
Prominent eustachian valve and chiari network	1
Multifenestrated ASD	3
Multiple defects	1
Large ASD (balloon occlusion diameter >30)	7

Abbreviation: ASD, atrial septal defect.

There was a success rate of 98.4%. Three cases failed. One of them had not TEE evaluation nor prior neither during the procedure because TEE was not available at that time. After this experience all of the subsequent cases were performed under guidance of TEE. The two other cases had deficient aortic rims associated with interatrial septal aneurysm in one and small superior rim in the other.

Mean hospital stay was 1.007 ± 0.0004 days. Complication rate was 7.42% (Table 3).

**Table 3:** Complications

**Complications**	**Number**
Death	0
Post-procedure large groin hematoma	2
Air emboli with transient ST elevation	3
Clot over LA disc	1
Late device malposition	1
Transient pericardial effusion	1
Transient recurrent PSVT	3
Transient frequent PVCs requiring therapy	1
Transient atrial fibrillation	2
Residual shunt (Qp/Qs) less than 1.5	3
Transient ischemic attacks	2

Abbreviations: PSVT, paroxysmal supraventricular tachycardia; LA, left atrial; PVC, premature ventricular contraction.

Our most common complications were transient arrhythmia including atrial fibrillation and recurrent paroxysmal supraventricular tachycardia, air embolism with transient ST elevation and residual shunt (Qp/Qs) less than 1.5.

Size of the RV annulus was significantly decreased 24 hours after intervention (Before intervention: 12.8±2.1 mm, after intervention 9.5±2.2 mm, *P*=0.005).

## Discussion

The present report demonstrates that transcatheter closure of ASD could be safe and effective. Procedural success rate was 98.8% which was comparable to prior studies. Three cases were failed in our study. One of them had not transesophageal echocardiographic evaluation nor prior neither during the procedure because TEE was not available at that time. After this experience all of the subsequent cases were performed under guidance of TEE. The two other cases had deficient aortic rims associated with interatrial septal aneurysm in one and small superior rim in the other. No cases of death or device embolization occurred.

The overall complications were 7.42%. Atrial arrhythmias occurred in 5 patients, 2 cases of atrial fibrillation (AF) and 3 cases of paroxysmal supraventricular tachycardia (PSVT) that all were successfully medically treated. There had been reports of these arrhythmias in previous studies.^11^ We had one patient with late device malposition that after 6 months the LA disk was malposed towards right atrium but without any left to right shunt. In three years of follow up of this patient, no other complications had been occurred. One of the serious complications occurred in a 17 years old female patient who developed thrombus formation on LA disk 2 weeks following device closure. It was confirmed via TEE and clinical and laboratory evaluations were performed to rule out infective endocarditis. Blood cultures were negative and all the inflammatory tests were normal. Anticoagulation with intravenous heparin followed by warfarin (target international normalized ratio [INR] around 2) was started and 2 months later the clot was completely resolved. There were not any further complications in 2.5 years follow up.

In our experience although the risk of complications were low (~7.4 %), it seems rational to follow some patients more closely, including (1) those with significantly larger amplatzer septal occluder (ASO) 1.5 times more than native diameter of ASD, (2) those with development of even small amount of pericardial effusion, (3) those with deformation of the ASO at the aortic root that could result in significant splaying of the device edges by the aorta, and (4) those with high defects (minimal aortic and superior rims) because patients with small aortic and superior rims are more prone to erosion and patients with small inferior rims are more prone to embolization and AV block.^13^

## Conclusion

Device closure of ASD is now a standard procedure for majority of secundum ASDs. The keys to have a good outcome are precise transesophageal echocardiographic evaluation, being familiar with all the possible complications and the knowledge to manage them.

## Ethical issues

Informed consents were taken from all the patients. This study was approved by the committee of ethics of Shiraz University of medical sciences.

## Competing interests

Authors declare no conflict of interest in this study.
